# *Bacillus* volatiles adversely affect the physiology and ultra-structure of *Ralstonia solanacearum* and induce systemic resistance in tobacco against bacterial wilt

**DOI:** 10.1038/srep40481

**Published:** 2017-01-16

**Authors:** Hafiz Abdul Samad Tahir, Qin Gu, Huijun Wu, Yuedi Niu, Rong Huo, Xuewen Gao

**Affiliations:** 1 Department of Plant Pathology, College of Plant Protection, Nanjing Agricultural University, Key Laboratory of Integrated Management of Crop Diseases and Pests, Ministry of Education, Nanjing 210095, PR China

## Abstract

Volatile organic compounds (VOCs) produced by various bacteria have significant potential to enhance plant growth and to control phytopathogens. Six of the most effective antagonistic *Bacillus* spp. were used in this study against *Ralstonia solanacearum (Rsc)* TBBS1, the causal agent of bacterial wilt disease in tobacco. *Bacillus amyloliquefaciens* FZB42 and *Bacillus artrophaeus* LSSC22 had the strongest inhibitory effect against *Rsc*. Thirteen VOCs produced by FZB42 and 10 by LSSC22 were identified using gas chromatography-mass spectrometry analysis. Benzaldehyde, 1,2-benzisothiazol-3(2 H)-one and 1,3-butadiene significantly inhibited the colony size, cell viability, and motility of pathogens and negatively influenced chemotaxis. Transmission and scanning electron microscopy revealed severe morphological and ultra-structural changes in cells of *Rsc.* Furthermore, VOCs altered the transcriptional expression level of *PhcA* (a global virulence regulator), type III secretion system (T3SS), type IV secretion system (T4SS), extracellular polysaccharides and chemotaxis-related genes, which are major contributors to pathogenicity, resulting in decreased wilt disease. The VOCs significantly up-regulated the expression of genes related to wilt resistance and pathogen defense. Over-expression of *EDS1* and *NPR1* suggest the involvement of SA pathway in induction of systemic resistance. Our findings provide new insights regarding the potential of antibacterial VOCs as a biocontrol tool against bacterial wilt diseases.

The phytopathogen *Ralstonia solanacearum* is a Gram-negative, soil-borne, aerobic, non-sporing, motile bacterium, which can cause bacterial wilt in a wide variety of potential host plants (approximately 450 crop species), especially in the *Solanaceae* family^1^,^2^,^3^,^4^. *Rsc* enters through the roots and produces an excessive amount of extracellular polysaccharides (EPS) within the vascular system, blocking the vascular tissue, causing wilt and ultimately leading to the death of infected plants in severe infections^5^. Bacterial wilt is a devastating disease of crops that occurs widely in tropical and subtropical regions of the world, resulting in severe economic loss^6^. Several studies have explored the control of soil-born plant diseases through plant growth promoting rhizobacteria (PGPRs), which have the ability to maintain plant pathogenic microbes below the threshold level in the soil^7^,^8^,^9^. A large number of biocontrol agents have been identified that manage the bacterial wilt pathogen, including *Bacillus* spp., *Pseudomonas* spp., *Trichoderma* spp., *Streptomyces* spp. and *Paenibacillus* spp^3^,^10^,^11^,^12^. *Bacillus* species are considered the most efficient species because they have the ability to produce spores that can survive in adverse environments^13^. Most of the processes involved in controlling phytopathogens through antagonistic microorganisms require physical contact among interacting partners as well as very close vicinity. However, many bacteria and fungi can inhibit other pathogenic fungi from a distance, which raises the possibility that these microorganisms must produce invisible volatile compounds that inhibit the growth of antagonistic organisms. Several recent studies have revealed that volatile organic compounds (VOCs) emitted by some antagonistic bacteria and fungi have antifungal or nematicidal properties^14^,^15^. Effmert (2012) reported 300 bacteria and fungi as VOC producers, and nearly 800 VOCs were recorded in the database of volatiles emitted by microorganisms (DOVE-MO). Among these, 671 VOCs belong to 212 bacterial species, and 335 belong to 96 species of fungi^16^.

Bacterial VOCs play a beneficial role in three ways: promoting plant growth, inhibiting the growth of plant pathogens and inducing systemic resistance^17^,^18^. Many bacterial VOCs have been reported to be plant growth promoters, such as 2,3-butanediol and acetoin by *Bacillus subtilis* GB03 and *B. amyloliquefaciens* IN937a, 2-pentylfuran by *B. megaterium* XTBG34, 13-tetradecadien-1-ol, 2-butanone and 2-methyl-n-1-tridecene by *Pseudomonas fluorescens* SS101^19^,^20^. In addition to their growth-promoting activity, the VOCs trigger plant tolerance to biotic^17^ and a-biotic factors^3^ by inducing systemic resistance. Ryu *et al*.^17^ first identified 2,3-butanediol from *B. subtilis* GB03 and *B. amyloliquefaciens* IN937a, which significantly induced resistance in the *Arabidopsis* plant against the pathogen *Pectobacterium carotovorum* sub sp. *carotovorum.* Similarly, *Paenibacillus polymyxa* E681 produced tridecane, which also induced systemic resistance in plants^21^. A large number of investigations, which were initiated in 1966, have reported that VOCs affect the growth and development of fungi^22^,^23^,^24^,^25^,^26^,^27^. However, studies examining the interactions between PGPR and phytopathogenic bacteria are in preliminary stages. The bacterial VOCs, 3-pentanol and 2-butanone, have been reported to induce resistance against the bacterial angular leaf spot pathogen, *Pseudomonas* s*yringae* pv. *lachrymans*^15^.

A few investigations have been reported regarding the mode of action of antimicrobial VOCs. However, there is evidence that VOCs damage the DNA of the pathogenic microorganism and induce a change in the expression level of ontogenesis-related enzymes, which may alter the growth of targeted organism^28^. Various studies have collectively revealed that the type three secretion systems (T3SSs), encoded by the hypersensitive response and pathogenicity (*hrp*) genes, T4SS pili, EPS and chemotaxis, are the major contributors to pathogenicity in *Rsc.* The type three secretion system is essential for the pathogenicity of *Rsc* because it facilitates the injection of type three effector proteins (TTE) into plant cells^29^,^30^,^31^,^32^. The transcriptional expression of virulence and a-virulence factors is controlled by a complex regulatory network, and *PhcA*, a global virulence regulator, is the center of this network^5^,^33^. The homolog of bacterial wilt resistance gene *RRS1* from *Arabdiopsis* was detected in tobacco, acting as major resistant gene against *Rsc*^34^. Similarly *RE-bw*, another homologue of bacterial wilt resistance gene *RRS1* was observed as resistant gene against bacterial wilt caused by *Rsc* in egg plant. Over-expression of *RE-bw* enhanced endogenous salicylic acid (SA) content and up-regulated the transcriptional expression of *EDS1, PAD4, NPR1* and *SGT1* which are the main components of SA signaling pathway^35^. In this context, we used six *Bacillus* spp. that have been shown to have effective antagonistic activity against plant pathogens. The main objective of this study was to evaluate the toxicity of VOCs produced by selected *Bacillus* spp. against *Rsc* and the effect on the activation of induced systemic resistance in tobacco against bacterial wilt. The toxicity of VOCs in this study was evaluated in terms of the effect on physiological, ultra-structural and virulence-related characteristics and also on the transcriptional expression of related genes (*PhcA*, T3SS, T4SS pili, EPS and chemotaxis). Similarly induction of systemic resistance was observed, investigating the transcriptional expression of disease resistance and defense related genes in tobacco.

## Experimental procedure

### Microorganisms and growth conditions

*Bacillus amyloliquefaciens* FZB42 was kindly provided by R. Borriss (ABiTEP GmbH, Berlin, Germany). All other strains of *Bacillus* and *Ralstonia* used in this study (see Supplementary material Table S1) were obtained from the Laboratory of Biocontrol and Bacterial Molecular Biology, Nanjing Agriculture University, Nanjing, China. *Bacillus* strains were grown in Luria-Bertani (LB) medium at 37 °C overnight and maintained in LB broth supplemented with 30% glycerol at −20 °C as working stocks. *Ralstonia solanacearum* TBBS1 was grown in tetrazolium chloride (TZC) agar medium (supplemented with 0.05% TTC)^36^ for 48 h at 28 °C and maintained in sterilized distilled water at room temperature. Single colonies with a pink center were transferred to Casamino Acid Peptone Glucose (CPG) agar medium for routine use^37^.

### Antibacterial activity evaluation of *Bacillus* spp

The agar diffusion method^38^ was used to test the antibacterial activity on CPG medium. A five-milliliter suspension of bacterial pathogen was mixed with150 ml of molten CPG medium (≤50 °C) after 18–24 h of shaking (200 rpm) at 28 °C, poured into plates and allowed to solidify. Three sterile filter papers (4 mm) were placed on the agar surface at equal distances, 2.5 cm apart from the center. Five μl cultures of *Bacillus* strains grown overnight were dropped on two of the filter papers, and the remainder was impregnated with 5 μl of sterile water as a control. The plates were sealed with Parafilm^®^ M (Pechiney, Neenah, WI, USA) and incubated at 28 °C for 48 hours, and the antibacterial effect was determined by measuring the diameter of the growth inhibition zones around the filter papers inoculated with *Bacillus* strains.

### Evaluation of the antibacterial activity of VOCs produced by FZB42 and LSSC2 against *Rsc*

*Bacillus amyloliquefaciens* FZB42 and *B. artrophaeus* LSSC22 were selected and further evaluated for volatile-mediated antibacterial activity against *Rsc* using the I-plate system^17^, which consisted of centrally partitioned plastic Petri dishes (85 × 15 mm) with no physical contact between the two microorganisms grown on either side. Ten μl of (18–24 h) *Rsc* culture (1 × 10^7^ CFU ml^−1^) grown in CPG broth was dropped in one partition with CPG agar medium, and the plates were sealed and incubated for 24 hours. After 24 hours, 10 μl of liquid overnight-grown culture of each *Bacillus* (1 × 10^8^ CFU ml^−1^) strain were dropped in the other partition containing modified minimal salt medium (MS) (1.5% agar, 1.5% sucrose, and 0.4% TSA (w/v)). The plates were double sealed and incubated at 28 °C for five days. Only the pathogen was inoculated on control plates. The diameter of *Rsc* measured in cm and viable cells of the pathogen were counted by the 10-fold serial dilution technique. Each experiment was performed with five replicates, and the experiment was repeated three times.

### Scanning and transmission electron microscopy (SEM & TEM)

External morphological changes of the pathogen cells were observed using Scanning Electron Microscopy (SEM), and ultra-structural changes in the pathogen cells were observed using Transmission Electron Microscopy (TEM) after exposure to the VOCs of FZB42 and LSSC22 for three days at 28 °C. For the preparation of pathogen cells for SEM and TEM, filter paper discs were removed from the plates containing pathogen colonies with and without exposure to the VOCs of biocontrol strains using flame-sterilized micro-tweezers. The cells were then collected directly into Eppendorf tubes, washed three times with sterile water and fixated with 2% glutaraldehyde (Solarbio, (Beijing) Co. Ltd.) for 30 min at 4 °C. Fixed cells were rinsed thrice for 10 minutes with 100 mM phosphate buffer, post-fixed for 3 h in 1% osmium tetroxide, followed by dehydration through an ethanol gradient^39^. Later, the samples were coated with gold and electron micrographs were obtained using the Hitachi Science System SEM (Hitachi S-3000N, Tokyo, Japan). Similarly, for TEM, cells were collected as mentioned above, and the samples were embedded in Epon 812 and sectioned with an ultramicrotome^40^. Ultra-structural changes in the cells were observed using a Hitachi transmission electron microscope (H-600, Hitachi, Tokyo, Japan).

### Swarming, swimming and twitching motility assays

The motility activity of *Rsc* exposed to volatile compounds from FZB42 and LSSC22 was assessed using divided Petri plates. *Rsc* cultures were normalized to an OD_600_ of 1.0, and then 2 μl of the cultures were spotted onto one compartment of the divided plates containing different percentages of agar in CPG medium for twitching and swarming motility, while for swimming motility, a sterile tooth pick was dipped and then touched gently in the center of a CPG agar medium compartment. Another compartment containing modified MS agar medium was spot inoculated with FZB42 and LSSC22 cell culture. The swarming and swimming motility halo were examined after incubation at 28 °C for 72 h, and twitching motility was observed after 48 hours. The experiment was repeated three times with five replicates each time. For swarming motility, 0.7% agar was used, for twitching motility, 1.6% agar was used, and for swimming motility, 0.2% agar was used.

### Chemotaxis assay

The chemotaxis assay was performed in divided plates with modified MS medium on one side and chemotaxis buffer medium on the other side (10 mM phosphate buffer, 0.1 mM EDTA, 1 μM methionine, 10 mM lactic acid, 0.35% agar and pH 7.3). Ten mm of the agar was removed from the medium, and the holes were refilled with 100 μl of host plant (tobacco) root exudates. *Rsc* was inoculated at a distance of 15 mm from the holes in one compartment, while FZB42 and LSSC22 cultures were spot inoculated in the other compartment as described above. The plates were sealed with Parafilm and incubated at 28 °C. The *Rsc* cells, which had moved towards the root exudates, were removed after three days, and the CFU/ml was counted on the CPG agar plates as described earlier. For the collection of root exudates, the tobacco seeds were surface sterilized and then transferred to 1% water agar plates. These plates were incubated at 4 °C overnight and then at 28 °C in the dark for 4 to 6 days. For each extraction, 40 germinated seedlings were transferred into a sterile 50-ml conical tube containing 5 ml of sterile chemotaxis buffer and incubated at 28 °C for 24 h. They were then filtered using a Millipore membrane filter (0.22 μm) and stored at −80 °C. This experiment was performed with five replicates and was repeated three times.

### Collection and analysis of VOCs by SPME/GC-MS

#### SPME Analysis

A 20-μl suspension of each *Bacillus* strain was inoculated into 30 ml of modified MS agar medium in a 100 ml-vial at 28 °C. To collect VOCs, 2 cm divinyl benzene/carboxen/PDMS (DCP, 50/30 μm) solid phase micro extraction (SPME) fiber (Supelco, Bellefonta, PA, USA) was used. Three days after incubation, the SPME fiber was inserted into the headspace of the vial containing bacteria and incubated at 50 °C for 30 min.

#### GC-MS analysis

GC-MS analysis was performed using a Bruker 450-GC gas chromatograph in combination with a Bruker 320-MS mass spectrometer. Helium gas was used as the carrier at a flow rate of 1 ml min^−1^. The SPME fibers were desorbed at 220 °C for 5 min, and GC–MS was run for 25 min. The starting temperature of the column was 35 °C for 3 min, which was increased to 180 °C at 10 °C/min and further increased to 240 °C at 4 °C/min, held for 5 min. The mass spectrometer was operated in the electron ionization mode at 70 eV with a source temperature of 220 °C, with continuous scanning from 50 m/z to 500 m/z. The mass spectra data for the volatile compounds were analyzed using the data in the NIST/EPA/NIH Mass Spectrum Library.

### Evaluation of the antibacterial activity of synthetic VOCs identified through GCMS analysis

Standard chemicals were purchased from Sigma-Aldrich or Aladdin, previously collected and analyzed through GC-MS analysis. They were tested as pure chemicals for their antibacterial volatile activity against *Rsc* using the I-plate system. Solid-state VOCs were dissolved in dimethyl sulfoxide (DMSO) to obtain a final concentration of 1 mg ml^−1^. Ten μl (1 × 10^7^ CFU/ml) of freshly grown *Rsc* (18–24 h) in CPG broth was dropped at the center of one half of the I-plate containing CPG agar, while on the other half, 100 μl of each of the tested VOCs or DMSO only was dropped. DMSO served as a control because it does not inhibit microorganisms. The plates were sealed and incubated for five days, and the results were measured as described earlier. Each experiment included three replicates and was repeated three times. Based on the results of the initial screening of individual VOCs, the most effective VOCs, BDH, 1,2-BIT and 1,3-BDN, were chosen for further detailed analysis regarding their antibacterial activity against *Rsc* at different concentrations to find minimum inhibitory content (MIC).

### Effect of the degree of concentration of VOCs

To test the effect of the concentration of VOCs on the growth inhibition of the pathogen, an experiment was designed using three partition plates with completely sealed portions without any air movement except holes in two walls of the partitions. This arrangement was established so that the VOCs moved from the first partition, encompassing either *Bacillus* or a synthetic chemical, to the second partition and then from the second partition to the third partition. The second and third partition both were inoculated with 10 μl of *Rsc* (18–24 h) culture. The entire experiment was repeated three times with five replicates per experiment.

### Effect of VOCs on the virulence and pathogenicity of *Ralstonia solanacearum* TBBS1

The pathogenicity assay of *Rsc* was performed after three days of exposure to FZB42 and LSSC22 VOCs, and then the *Rsc* cells were harvested in sterile distilled water to an OD of 0.1 at 600 nm. Five tobacco plants were inoculated at the three to four leaf stage by puncturing the stem at the third fully expanded leaf from the apex with the help of an inoculum-dipped needle, and then 100 μl of the VOC-treated *Rsc* suspension (1 × 10^7^ CFU ml^−1^) was injected into each plant stem^41^. Similarly, 5 plants were inoculated with sterile water, and 5 plants were inoculated with a suspension of *Rsc* (1 × 10^7^ CFU ml^−1^) cells that were not exposed to VOCs. The experiment was performed using a completely randomized design and repeated three times with five replicates in each experiment. The data for wilt was collected 21 days after inoculation using the following formula: Disease index (%) = [*Σ (ni* × *vi*) ÷ (*V* × *N*)] × 100, where *ni* indicates the number of plants with the respective disease rating; *vi* = disease rating; *V* = the highest disease rating (5) and *N* = the number of plants observed. The disease rating was calculated using the scale: 1 = no symptoms, 2 = one leaf wilted, 3 = two to three leaves wilted, 4 = four or more leaves wilted and 5 = whole plant wilted.

### Pathogen inoculation and effect of VOCs on wilt disease development

The experiment was divided into two parts, i.e., the determination of the effect of VOCs on wilt disease in Petri plates and in plastic pots fitted on jars. I-plates prepared with one-half-strength Murashige and Skoog solid media and 5–6-day-old emerging tobacco seedlings (seven seedlings/plate) were dipped in the suspension of *Rsc* (1 × 10^7^ CFU/ml) cells and then transplanted into one compartment. The non-inoculated control roots were dipped in sterile water. Bacterial strains, FZB42 and LSSC22, were cultured on LB broth one day before as described above. A 20-μl (10^8^ CFU/ml) suspension of each strain or synthetic chemical was dropped into the other compartment. For individual chemical assay, each chemical with two different concentrations i.e. 10 mM 1,3-BDN and 1 mM 1,3-BDN, 1 mM 1,2-BITH, 0.1 mM 1,2-BITH, 0.1 mM BDH and 0.01 mM BDH were used in one compartment, while in other compartment tobacco seedlings as described earlier. Wilt symptoms were observed after one week of inoculation, and the data were recorded as described earlier. In the other experiment, plastic pots were fixed on glass jars (60 × 120 mm) and sealed with Parafilm to avoid the escape of VOCs from the jar, and a Petri dish (35 × 12 mm) was inoculated with FZB42, LSSC22 or sterilized water (control) and placed at the bottom of the jar. Filter paper was used at the bottom of the plastic pot containing tobacco seedlings to ensure that only VOCs produced by FZB 42 and LSSC22 could come into contact with the tobacco plants under soil conditions. Eight small holes (4 mm) were created at the bottom of the plastic pot for VOCs exposure. All pots were inoculated with a suspension of *Rsc* (at an OD of 0.1 at 600 nm) by dipping the roots in the suspension, except the non-inoculated control, and then the seedlings were re-planted in the pots. The inoculated seedlings were kept in a growth chamber at 28/22 °C day/night temperature for a 16/8-h light/dark photoperiod at 85% relative humidity. The experiment was performed using a completely randomized design with five replicates, and the whole experiment was repeated three times. The data for wilt was collected 21 days after inoculation using the formula mentioned above.

### Real-time PCR

The *Rsc* cells were harvested after exposure to VOCs from FZB42 and LSSC22 for 72 hours. Total RNA was extracted using the Bacterial RNA Kit (Omega Bio-Tek, Norcross, GA, USA) according to the manufacturer’s instructions. First-strand cDNA was obtained using Reverse Transcriptase (TaKaRa Bio Inc., Tokyo, Japan) with random hexamer primers. The 16srRNA was used as an internal reference, and the transcriptional levels of *PhcA,* TSSS, Type IV pili, EPS and chemotaxis related genes were detected. For tobacco plants, leaves were collected at 3^rd^, 6^th^ and 9^th^ days after inoculation with *Rsc*. Total RNA was isolated using the TRIzol reagent (Invitrogen Biotechnology Co., Carlsbad, CA, USA) according to the manufacturer’s instructions. The *EF-1α*^42^ was used as an internal reference, and the transcriptional expression levels of *RRS1, NPR1* and *EDS1* genes were detected. Real-time PCR was performed using a SYBR Green/Fluorescent qPCR master mix (Takara) on a Roche-480 system (Roche). The qRT-PCR program consisted of denaturation at 95 °C for 1 min, followed by 40 amplification cycles at 95 °C for 5 sec, 57 °C for 30 sec, and 72 °C for 30 sec. The specific primers used in this study are listed in Table S2. Each sample was replicated thrice for qPCR, and 2−ΔΔCt method was used to analyze gene expression level^43^.

### Statistical analysis

To evaluate the significance of the treatments, the data from each experiment was analyzed using analysis of variance (ANOVA) and Duncan’s multiple-range test was employed to assess differences among treatments at *P* = 0.05 using SPSS ver. 17.0 statistical software (SPSS, Chicago, IL). Graphs and figures were plotted using sigma plot version 10.0.

## Results

### Evaluation of antibacterial activity

**Figure 1:**
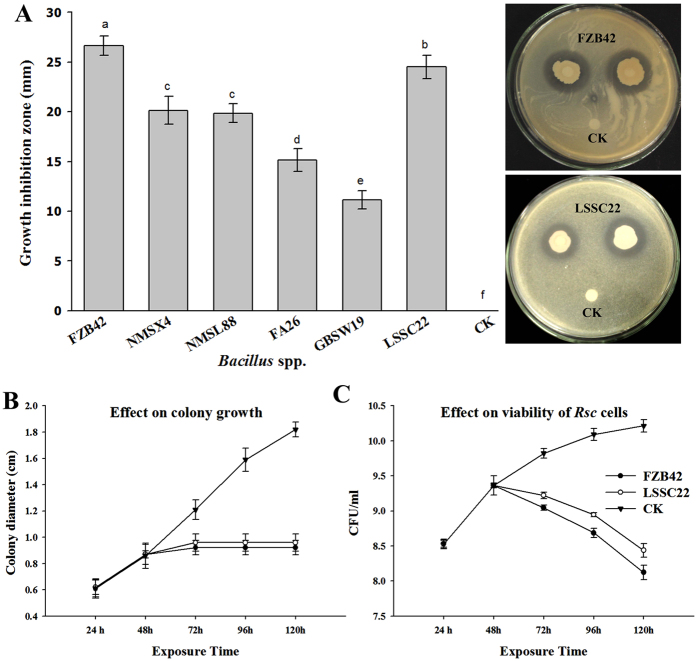
The effect of strains of *Bacillus* on the growth of *Rsc*. (**A**) The mean growth inhibition zones (mm) produced by antagonistic *Bacillus* spp. (**B**) Antibacterial volatile activity of *B. amyloliquefacians* FZB42 and *B. artrophaeus* LSSC22 against *Ralstonia solanacearum*. Error bars indicate standard deviations of the three replicates. Different letters above error bars represent significant differences according to Duncan’s multiple-range test (P = 0.05) using SPSS software (SPSS, Chicago, IL).

All antagonistic strains inhibited the growth of *Rsc* at varying levels, but *Bacillus amyloliquefacieans* FZB42 produced the largest zone of inhibition (26.65 mm ± 1.01 SD), followed by *Bacillus artrophaeus* LSSC22 (24.51 mm ± 1.55 SD). *Bacillus amyloliquefacians* NMSX4 and *B. cereus* NMSL88 inhibited the pathogen at the same level (20.15 mm ± 1.39 SD and 19.85 mm ± 0.95 SD) and were not significantly different. *Bacillus pumulis* GBSW 19 produced the smallest zone of inhibition of 11.15 mm ± 0.94 SD (Fig. 1A). FZB42 and LSSC22, which was shown to be most effective against *Rsc* in the GIZ experiment, were further evaluated *in vitro* for the production and activity of antibacterial VOCs against the pathogen *Rsc.* FZB 42 inhibited the colony growth of *Rsc*, restricting to 0.92 cm ± 0.055 SD and LSSC22 to 0.96 ± 0.065 SD cm as compared to control 1.82 cm ± 0.057 SD (Fig. 1B). Inhibition in colony diameter was 49.39% by FZB42 and 47.25% by LSSC22. The number of viable cells of *Rsc* also decreased when exposed to VOCs produced by FZB42 and LSSC22 and this decrease was maximum at 120 h (Fig. 1C).

### *Bacillus* VOCs caused morphological and ultra-structural abnormalities in *Rsc* cells observed by SEM and TEM

**Figure 2:**
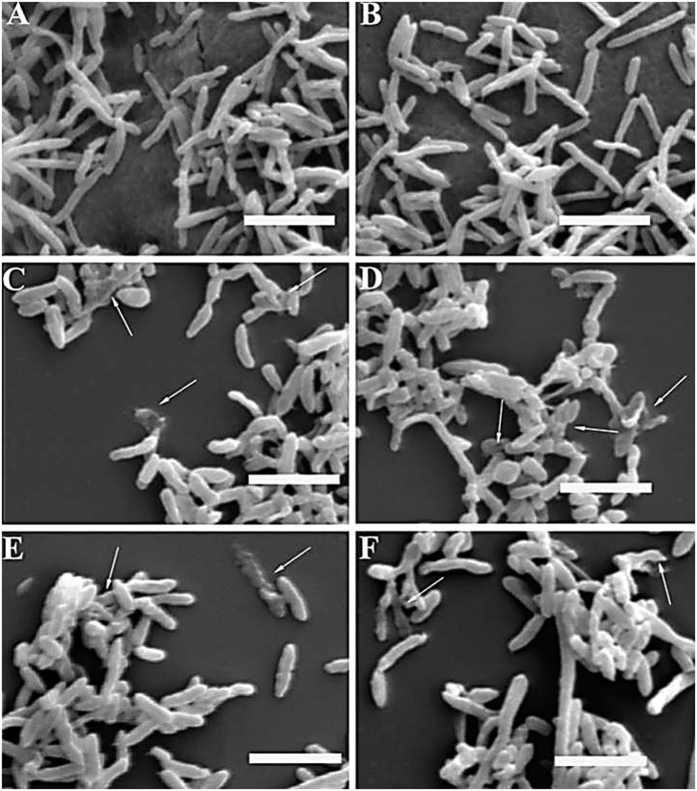
*Bacillus* VOCs caused a wide range of abnormalities in the morphology of the *Ralstonia solanacearum*cells observed by SEM. Pathogenic cells were grown on CPG at 28 °C, and after three days of exposure to VOCs of FZB 42 and LSSC22, samples were collected from the I-plates. Electron micrographs were obtained using a Hitachi Science System SEM (Hitachi S-3000N, Tokyo, Japan). In the control (water), *Rsc* was grown on CPG without exposure to VOCs. The colony morphology of the pathogen was altered in the presence of volatiles produced by FZB 42 **(C,D)** and LSSC22 **(E,F)** and revealed several deformed pathogenic cells within the colony of the pathogen compared with the control **(A,B).** Arrow heads indicating distortion of *Rsc* cells. Bars; 5 μm.

**Figure 3:**
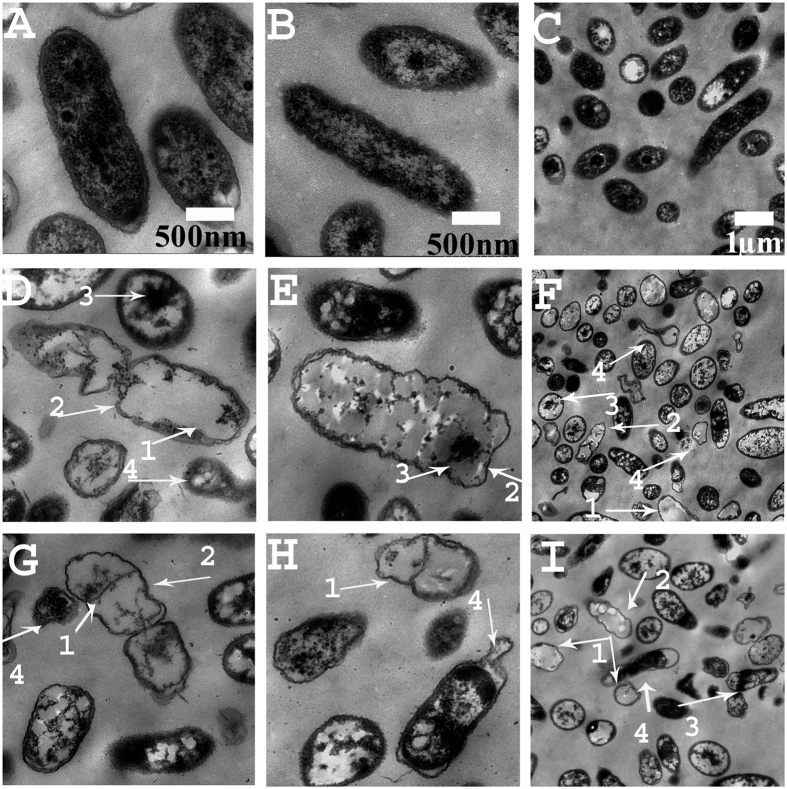
*Bacillus* VOCs caused severe ultra-structural abnormalities in the *Ralstonia solanacearum* cells observed by TEM. *Ralstonia solanacearum* cells were collected as previously mentioned and were prepared for TEM. Ultra-structural changes in the cells were observed using a Hitachi transmission electron microscope (H-600, Hitachi, Tokyo, Japan). Compared with the undamaged cells in the control **(A–C)**, pathogenic cells showed a wide range of abnormalities after exposure to the VOCs of FZB42 **(D–F)** and LSSC22 **(G–I)**. These changes included **1.** Movement of cytoplasmic contents, **2.** Loosening of the cell wall, **3**. Formation of inclusion bodies and **4**. Misshapen bacteria.

TEM and SEM analysis revealed a wide range of abnormalities in pathogenic cells that were affected by FZB 42 and LSSC22 when compared to the untreated control. SEM of the non-treated samples showed normal growth (Fig. 2A,B), whereas the colonies of pathogenic cells were severely disrupted following treatment with VOCs, as shown in Fig. 2C–F. The ultra-structural changes due to antibacterial VOCs of these *Bacilli* on the pathogenic cells of *Rsc* were further observed using TEM. Transmission electron micrographs of the *Rsc* cells obtained from the non-inoculated control showed apparently intact envelops with evenly distributed electron-dense cytoplasmic contents (Fig. 3A–C). The pathogenic cells exposed to VOCs of FZB42 and LSSC22 exhibited a large number of abnormalities. These abnormalities included loosening, and even at some places rupturing, of the cell wall, movement of the cytoplasmic content towards these ruptured wall areas, in some cells a lack of differentiated materials in the cytoplasm and also misshapen bacterial cells (Fig. 3D–I).

### *Bacillus* VOCs inhibited motility (swarming, swimming and twitching) and chemotaxis of *Rsc*

**Figure 4:**
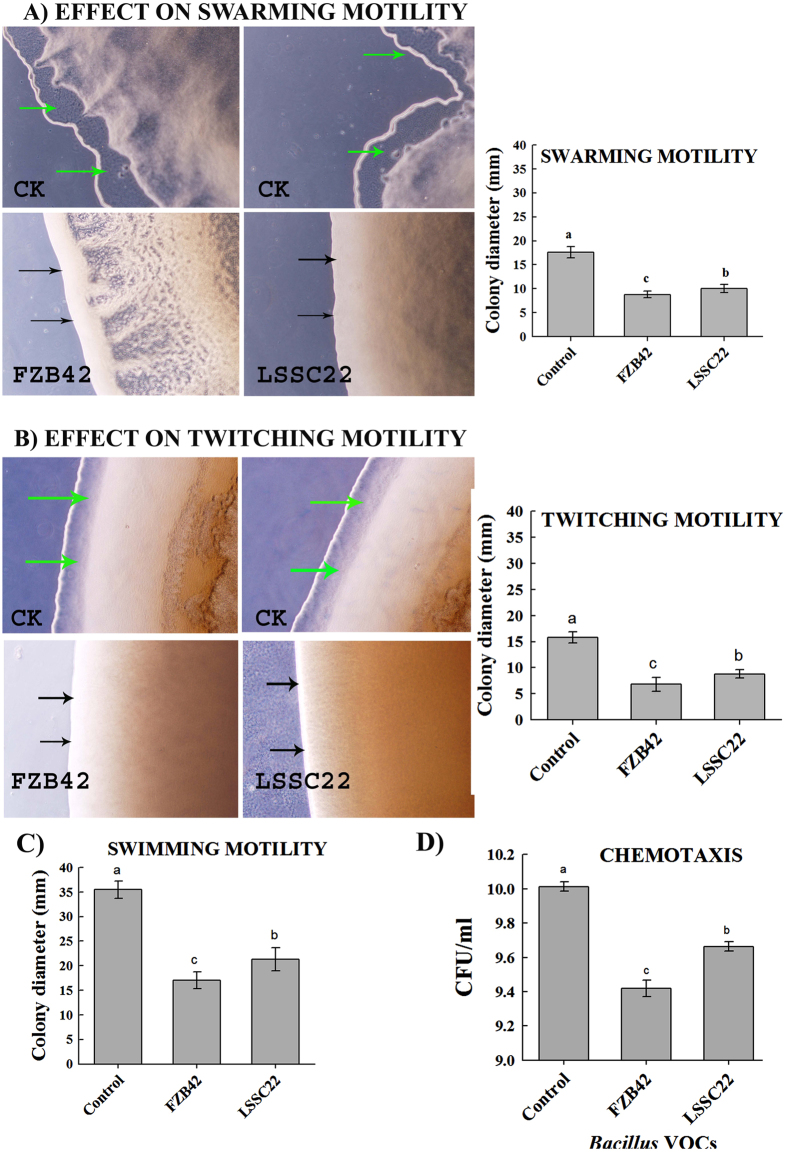
Effect of VOCs produced by *Bacillus amyloliquefacians* FZB42 and *Bacillus artrophaeus* LSSC22 on motility of *Ralstonia solanacearum*. (**A**) Swarming motility (**B**) twitching motility (**C**) swimming motility (**D**) chemotaxis Error bars indicate standard deviations of the means. The data were analyzed using analysis of variance (ANOVA) followed by Duncan’s multiple-range test (P = 0.05) using SPSS software (SPSS, Chicago, IL). Error bars indicate standard deviations of three replicates and different letters describe significant differences at P = 0.05 within the same data group.

The volatile compounds produced by FZB42 and LSSC22 had a negative effect on the motility of *Rsc.* VOCs produced by FZB 42 significantly reduced all three motility types. Swarming motility was restricted to 8.80 mm by FZB42 and 10.00 mm by LSSC22 as compared to control 17.60 mm, while swimming motility was restricted to 17.00 mm by FZB42 and 21.30 mm by LSSC22 as compared to control 35.00 mm. Similarly twitching motility was limited to 6.80 mm by FZB42 and 8.80 mm by LSSC22 as compared to control 15.80 mm (Fig. 4A–C). The inhibition in motility by FZB42 was 50%, 52.11% and 56.96% in swarming, swimming and twitching, respectively, compared with the non-exposed control. Swarming motility was reduced by 43.18%, swimming by 40% and twitching by 44.30%, compared with the control following exposure to VOCs produced by LSSC22. In the chemotaxis assay, *Rsc* showed significantly greater motility toward the hole containing root exudates in the control. FZB 42 and LSSC22 inhibited the chemotaxis of *Rsc* by 74.50% and 56.13%, respectively (Fig. 4D). In the negative control, there was no directed motility in any direction, but slight motility of *Rsc* cells in all directions was observed because sterile water was used instead of root exudates. The negative control justified that chemotaxis was present in *Rsc* along with motility.

### GC-MS analysis of VOCs produced by FZB42 and LSSC22

Volatiles produced by FZB42 and LSSC22 were collected by a combination of HS-SPME and GC-MS. The volatile fractions of the antagonistic bacteria were compared with the volatiles retrieved from the control (non-inoculated medium). Mass spectra data of the volatile compounds were analyzed using the data in the NIST/EPA/NIH Mass Spectrum Library. Thirteen compounds were identified from FZB42 and 10 compounds were identified from LSSC22 (see supplementary material, Table S3), which had relatively high peak areas, e.g. ≥1%, and were not similar to the control. 1,2-Benzisothiazol-3(2 H)-one (1,2-BIT), (1 R)-2,6,6-Trimetyhlbicyclo[3.1.1]-hept-2-ene (TMB), Benzoic acid (BA), Dodecane, 1-fluoro (DCF), Dodecane (DCN) and Phenol, 2-(1,1-dimethylethyl)-6-methyl (PH) were common both in FZB42 and LSSC22. Seven other VOCs: Silanediol, dimethyl (SDD), Benzeneacetamide (BAM), Oxime, methoxy-phenyl (OMP), Benzaldehyde(BDH), Sulfurous acid, cyclohexyl-methyl isobutyl ester (SCE), 6-Tridecen, 2,2,4,10,12,12-hexamethyl-7-(3,5,5-trimrthylhexyl)-(6THT) and 2-Undecanethiol, 2-methyl (2-UT,2-M) were found in FZB42 while four VOCs: 1,3–Butadiene (1.3BDN), 1-octyn-3ol, 4-ethyl-(1OCTN), Undecanal, 2-methyl (UDM) and Cyclohexene, 3-(1,5-dimethyl-4-hexenyl)-6-methylene-(CHN) were found in LSSC22.

### BDH, 1,2-BITH and 1,3-BDN had strong antibacterial activity against *Rsc*

**Figure 5:**
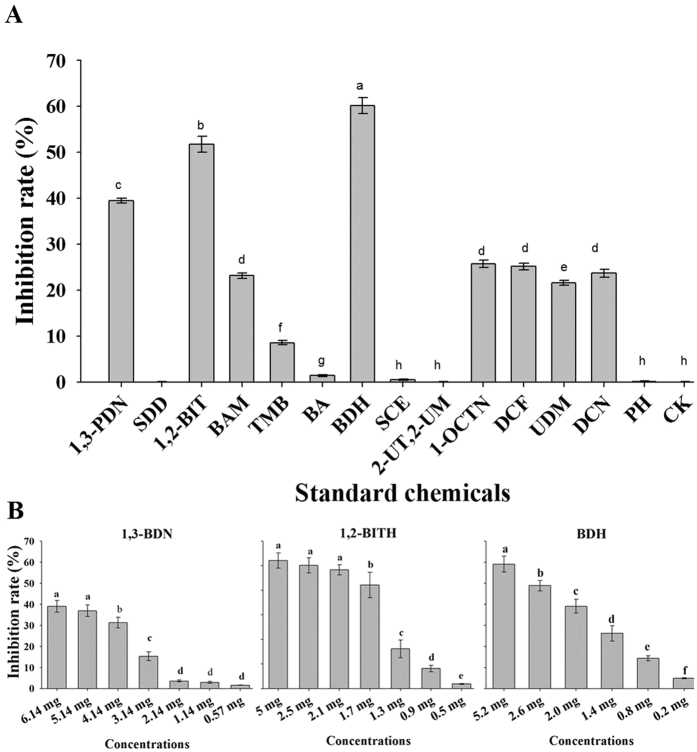
BDH, 1,2-BITH and 1,3-BDN showed strong antibacterial activity against *Ralstonia solanacearum*. The data were analyzed using analysis of variance (ANOVA) followed by the Duncan’s multiple-range test (P ≤ 0.05) using SPSS software (SPSS, Chicago, IL). Error bars indicate the mean ± SD and different letters describe significant differences at P = 0.05 within the same data group. The compounds are ordered according to their retention time on a capillary GC column (Supelcowax^®^10). **1,3-BDN** = 1,3–Butadiene, **SDD** = Silanediol, dimethyl, **1,2-BIT** = 1,2-Benz isothiazol-3(2 H)-one, **BAM** = Benzeneacetamide, **OMP** = Oxime, methoxy-phenyl, **TMB** = (1 R)-2,6,6-Trimetyhlbicyclo[3.1.1]hept-2-ene, **BA** = Benzoic acid, 2-formyl-4,6-dimethoxy-,8,8-dimethoxyoct-2-yl, **BDH** = Benzaldehyde, **SCE** = Sulfurous acid, cyclohexylmethyl isobutyl ester, **2-UT,2-M** = 2-Undecanethiol, 2-methyl, **1,OTN** = 1-octyn-3ol, 4-ethyl-, **DCF** = Dodecane, 1-fluoro, **UDM** = Undecanal, 2-methyl **DCN** = Dodecane, **PH** = Phenol, 2-(1,1-dimethyl)-5-methyl.

The results showed that three chemicals, BDH, 1,2-BITH and 1,3-BDN, reduced the viability of the *Rsc* cells by 60.113%, 51.65% and 39.89%, respectively, compared with the control. These chemicals were selected for further study at different concentrations to find minimum inhibitory content (MIC). SDD, SCE, 2-UT2-M and PH did not inhibit the growth of *Rsc*, while BAM, 1-OCTN, DCF, UDM and DCN had a moderate effect, resulting in a 23.11%, 25.73%, 25.15%, 21.54% and 23.66% inhibition rate, respectively. TMB and BA had the least inhibition (Fig. 5A). BDH, 1,2-BITH and 1,3-BDNwere further evaluated at different concentrations, which showed that BDH, 1,2-BITH and 1,3-BDN reduced the viable cells even at 0.20 mg, 0.50 mg and 0.57 mg, respectively (Fig. 5B).

### Antibacterial activity of VOCs was dependent on the concentration of VOCs

The results showed that after five days of incubation, the 2^nd^ partition had more growth inhibition in the form of less viable cells of *Rsc* than the 3^rd^ partition following inoculation with FZB42. Similar results were obtained when synthetic VOC 1,2-BITH was used. However, the most effective VOC, BDH (undiluted), inhibited the growth of *Rsc* completely in both partitions, while the control did not inhibit growth (see Supplementary material Fig. S1).

### *Bacillus* VOCs altered the transcriptional expression of T3SS, T4SS pili, EPS and chemotaxis-related genes in *Rsc*

**Figure 6:**
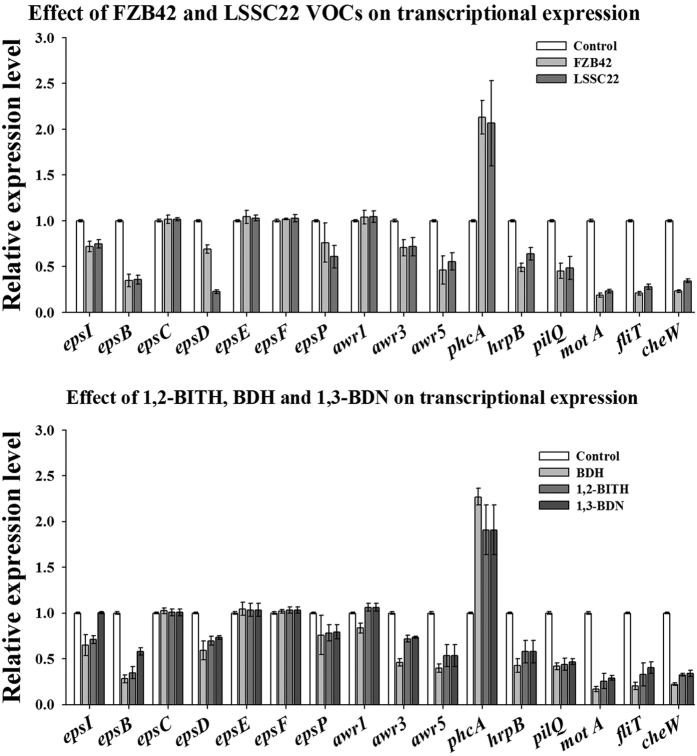
Transcriptional expression of virulence-related genes in *Ralstonia solanacearum*. The *Rsc* cells were harvested after exposure to VOCs from *B. amyloliquefacians* FZB 42 and *B. artrophaeus* LSSC22, benzaldehyde, 1,2-benz isothiazol-3(2 H)-one and 1,3-butadiene (1,3-BDN) for 72 hours. Real-time PCR was performed using a SYBR Green/Fluorescent qPCR master mix (Takara) on a Roche-480 system (Roche). The 16srRNA was used as an internal reference, and the transcriptional levels of *PhcA*, T3SS, T4SS pili, EPS and chemotaxis*-*related genes were detected. Each sample was replicated thrice for qPCR, and 2−ΔΔCt method was used to analyze gene expression level. Error bars indicate standard deviations of the means of three replicates.

The transcriptional expression of genes related to extracellular polysaccharides (*epsI, epsB, epsC, epsD, epsE, epsF, epsP*), motility (*motA, fliT*), T3SS (*hrpB*), TTE (*awr1, awr3, awr5*), T4SS pili (*pilQ*), chemotaxis (*cheW*), and the global virulence regulator, *PhcA*, were significantly altered after exposure to VOCs of FZB42, LSSC22 and synthetic chemicals. Real-time PCR analysis elucidated that, of the 16 genes examined, 11 (*epsI, epsB, epsD, epsP, motA, fliT, hrpB, awr3, awr5, pilQ*, and *cheW*) were down-regulated at various levels, one (*phcA*) was up-regulated, and 4 (*epsC, epsE, epsF* and *awr1*) were unaffected after *Rsc* cells were exposed to VOCs of FZB 42 and LSSC22 for three days at 28 °C. BDH down-regulated 12 genes, including *awr1*, which was unaffected by FZB42 and LSSC22. Similarly, 1,2-BITH and 1,3-BDN altered the expression levels of 12 genes (*epsI, epsB, epsD, epsP, awr3, awr5, phcA, hrpB, pilQ, motA, fliT*, and *cheW*). However, 1,3-BDN had no effect on *epsI* along with four other unaffected genes (Fig. 6).

### *Bacillus* VOCs reduced the virulence and pathogenicity of *Rsc* and induced systemic resistance

**Figure 7:**
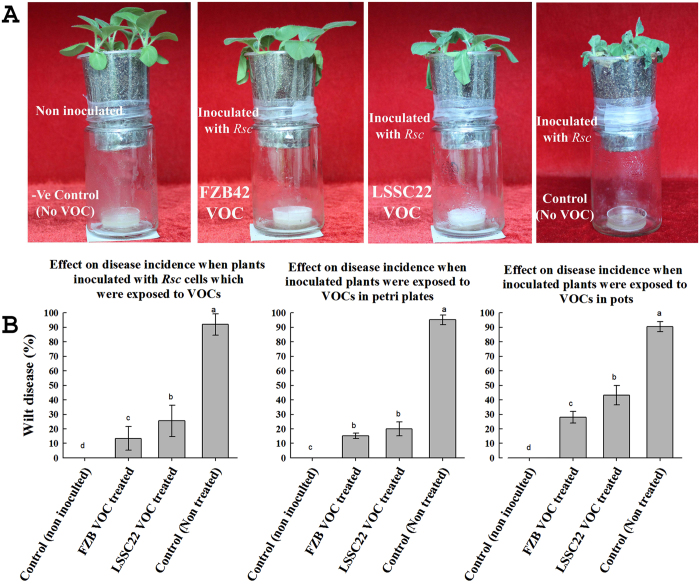
Volatiles produced by *B. amyloliquefacians* FZB42 and *B. artrophaeus* LSSC22 reduced the virulence of *Ralstonia solanacearum*and and induced systemic resistance. %Wilted plants was calculated after 21 days of inoculation according to the Winstead formula. Five tobacco plants were inoculated at the three to four leaf stage by puncturing the stem at the third fully expanded leaf from the apex with the help of an inoculum-dipped needle. Then, 100 μl of the VOC-treated *Rsc* suspension was injected into each plant stem. In the control, plants were inoculated with sterile water, and in the non-treated group, plants were inoculated with a suspension of *Rsc* cells that were not exposed to VOCs. For the Petri plate experiment (B2), 5-6-day-old emerging tobacco seedlings inoculated with *Rsc* were transplanted into one compartment, bacterial strains were cultured in other compartment of I-plate. In the non-inoculated control, the roots were dipped in sterile water. Wilt symptoms were observed after 7 days of inoculation, and the data were recorded as described earlier. In B3, plastic pots, fixed on glass jars, having a Petri dish inoculated with FZB42, LSSC22 or sterilized water were used. All three pots were inoculated with a suspension of *Rsc* by the root dip method, except the non-inoculated control, and were kept in a growth chamber at 28/22 °C day/night temperature for a 16-h light/8-h dark photoperiod at 85% relative humidity. Error bars indicate standard deviations of the means. Different letters above error bars represent significant differences according to Duncan’s multiple-range test (P = 0.05) using SPSS software (SPSS, Chicago, IL).

**Figure 8:**
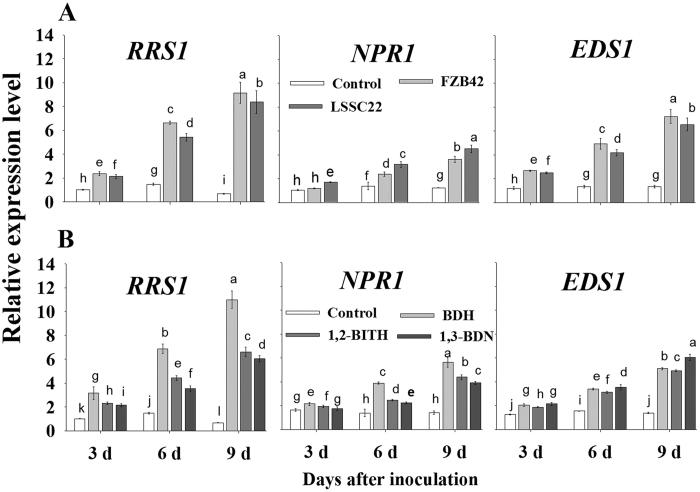
Transcriptional expression of virulence-related genes in *Ralstonia solanacearum*. Relative expression of the resistance gene *RRS1* and defense related genes *NPR1* and *EDS1* in tobacco were observed after exposure to VOCs produced by FZB42 and LSSC22 (**A**) and synthetic chemicals (**B**) in pot experiment. Error bars indicate standard deviations of three replicates and 2−ΔΔCt method was used to analyze gene expression level.

The pathogenicity assay of *Rsc* was performed after three days of exposure to VOCs of FZB42 and LSSC22, which resulted in a decrease in the virulence of *Rsc.* A 13.33% wilt disease index was observed when *Rsc* was exposed to VOCs from FZB42, and a 25.45% index was observed for LSSC22, while the index was 92.00% when *Rsc* cells were not exposed to any bacterial VOCs. FZB42- and LSSC22-derived VOCs reduced disease development significantly (by 15.2% and 16%, respectively) when tobacco seedlings were exposed to their VOCs, compared with the control (95%), but there was no significant difference between the treatments. Similarly BDH, 1,2-BITH and 1,3-BDN also reduced the disease development. A 14.4% and 15.2% disease index was observed by BDH (0.1 mM and 0.01 mM), 18.4 and 20.8% by 1,2-BITH (1 mM and 0.1 mM) while 24.8 and 28. % by 1,3-BDN (10 mM and 1 mM) as compared to (97.6%) water control. Reduction in disease development in the I plate system confirmed the induced systemic resistance in tobacco against *Rsc* by VOCs of FZB42 and LSSC22 as well as by synthetic chemicals (see Supplementary material Fig. S2). To demonstrate that the VOCs produced by FZB42 and LSSC22 induced systemic resistance at a broader level (*in-vivo*), we created the *in planta* system. The results showed that VOCs produced by FZB42 and LSSC22 caused a significant reduction in the wilt index, confirming the induced systemic resistance in plants against *Rsc*. FZB42 significantly reduced the disease and showed only a 28% wilt index, while LSSC22 had a 43.20% wilt index, as compared with a 92% wilt index in the non-exposed control (Fig. 7). To verify the resistance in tobacco against *Rsc,* due to the exposure of VOCs in pot experiment, we analyzed the genes of tobacco plant relating to resistance and defense by real time PCR. The transcriptional expression of R gene *RRS1* which is related to resistance in tobacco against *Rsc* was induced by FZB42, LSSC22 and also by individual chemicals; BDH, 1,2-BITH and 1,3-BDN as compared to untreated control. However over-expression was more by FZB42 and BDH as compared to others and increased with the time, showing its maximum at 9^th^ days after inoculation. An up-regulation in defense related genes *NPR1* and *EDS1* was observed after exposure to *Bacillus* VOCs. However, there was a difference in the relative expression levels of *NPR1* and *EDS1* among the VOCs produced by FZB42 and LSSC22. Increased expression of *NPR1* was noticed when treated with VOCs of LSSC22 compared with FZB42, which reached the highest level at the 9th day. Expression of *EDS1* was also induced by FZB42 and LSSC22-VOCs (Fig. 8A). Similarly, synthetic VOCs BDH, 1,2-BITH and 1,3-BDN induced the transcriptional expression of genes relating to defense at varying level as compared to control (Fig. 8B). These results indicate that VOCs stimulated the resistance and defense related genes which activated the induction of systemic resistance in tobacco against *Rsc*.

## Discussion

Microbial volatiles have been increasingly studied during the last two decades for their beneficial and environmentally friendly roles, such as induced systemic resistance against plant pathogens and abiotic factors^21^,^44^, plant growth enhancement^15^,^20^ and antagonistic action against plant pathogenic fungi and nematodes^8^,^14^. However, most investigations have been conducted using plant pathogenic fungi^15^. Recently, antibacterial activity of VOCs produced by *Bacillus amyloliquefaciens* SQR-9 and *Pseudomonas fluorescens* WR-1 was demonstrated against the tomato wilt pathogen, although this study did not further investigate the induction of systemic resistance against *Rsc*^45^,^46^. In our study, we demonstrated that bacterial VOCs not only control bacterial pathogens but can also induce systemic resistance. *Bacillus amyloliquefaciens* FZB42 is well known for plant growth regulation and the synthesis of complex bioactive molecules such as microcin B17, streptolysin S and amylocyclicin that inhibit the growth of plant pathogenic fungi and bacteria^47^,^48^,^49^. *Rsc* requires swimming motility and twitching motility for virulence^31^,^50^, as non-motile mutants of *Rsc* have been demonstrated to be significantly less virulent. There is a strong relationship between swimming motility and virulence, as swimming motility is a virulence trait of *Rsc* and is required for the wilt pathogen to properly invade and colonize the host plant^50^. *Rsc* not only requires motility but also chemotaxis toward root exudates to efficiently colonize and enter into plant roots for disease development^51^. Our results revealed a reduction in both motility and chemotaxis of *Rsc* following exposure to VOCs of FZB42 and LSSC22 (Fig. 4). This phenomenon was confirmed in the pathogenicity assay and the transcriptional expression of motility-related genes. Twitching motility is a form of bacterial translocation over firm surfaces that requires T4SS pili. Our results revealed down-regulation of *pilQ, fliT, motA* and the chemotaxis-related gene *cheW* (Fig. 7). T3SS, T4SS pili, EPS and chemotaxis are major pathogenicity determinants of *Rsc*. In *Rsc,* the expression of Hrp-T3SS-related genes is regulated by an AraC-type transcriptional activator, *HrpB*, which is down-regulated by VOCs from FZB42 and LSSC22. Thirty HrpB-regulated hpx (hrpB-dependent expression) genes and three hrpB-regulated genes, *popA, popB* and *popC*, have been identified in *Rsc*^32^. *HrpB* acts as a master regulatory gene, and the down-regulation of *hrpB* might ultimately affect these genes. T3SS encoded by hypersensitive response and pathogenicity (hrp) genes delivers bacterial effector proteins called type three effector proteins (TTE) directly into the host cell cytosol to promote disease^30^,^52^. AWR (alanine-tryptophan-arginine tryad) effectors are involved in virulence or avirulence in *Rsc* upon interaction with the host plant. Our results showed down-regulation of *awr3* and *awr5*. Transcriptional expression of *awr* (type III effecter proteins) requires *HrpB*^53^ which was down-regulated in our study, justifying the down-regulation of *awr3* and *awr5*. A complex regulatory network is required for the transcriptional expression of virulence or avirulence factors, and PhcA is the control center of this system^33^. PhcA is directly or indirectly involved in the regulation of genes, resulting in the expression of several virulence factors such as EPS, plant cell wall-degrading enzymes, TTSS and bacterial motility^54^. Results showed that transcriptional expression of *PhcA* was up-regulated up to 3-fold following treatment with VOCs of FZB42 and LSSC22. The global virulence regulator, *PhcA*, negatively regulates the expression of T3SS genes, supporting our results. Similarly, Hrp pili in *Rsc* were also repressed by *phcA* in cells grown in rich medium^54^. VOCs from FZB42 and LSSC22 repressed *epsI, epsB, epsD* and *epsP*, while *epsC, epsE* and *epsF* were not affected. EPS is the one of the most important virulence factors because EPS I-deficient mutants are nearly avirulent and do not colonize plant xylem vessels as efficiently as wild-type pathogens^5^. Our results are in conformity with previously reported results in which repressed transcriptional expression of virulence-associated genes in *Xanthomonas oryzae* pv. *oryzae* were noted^55^. Similarly VOCs produced by *Bacillus amyloliquefaciens* SQR-9 and *Pseudomonas fluorescens* WR-1 down regulated the expression of *RSc* proteins related to virulence^46^,^47^. At high concentrations, VOCs might damage the cell membrane, causing the movement of intracellular material out of the cell and resulting in cell death. Electron micrographs of the untreated *Rsc* samples revealed normal growth of the cells, while the *Rsc* colonies were disturbed, in the presence of VOCs of FZB42 and LSSC22. The transmission electron micrograph showed that VOCs caused damage to the pathogenic cells at high concentrations, while the non-inoculated control displayed normal cell growth. These abnormalities included loosening of the cell wall and, in some cells, rupturing of the cell wall and movement of the cytoplasmic contents towards the ruptured walls, resulting in the formation of misshapen cells and sometimes cell death (Figs 2 and 3). These results are consistent with previously reported studies in which altered morphology of fungal hyphae and conidiophores of *Botrytis cinerea* and *Penicillium italicum* were observed after exposure to microbial VOCs^56^. Similarly SEM and TEM analysis showed abnormal surface morphology in cells of *Xanthomonas oryzae* pv. *oryzae* after exposure to VOCs produced by *Bacillus cereus* D13^55^. One prominent finding of our study was that VOCs from FZB42 and LSSC22 negatively affected disease development and induced systemic resistance in tobacco plants. However, disease development appeared to be greater in the *Rsc* inoculated plants that were treated directly with VOCs. This observation might be due to the hindered contact of VOCs with *Rsc* cells (Fig. 7B1 and B3). The tobacco seedlings grown in divided Petri plates displayed a greater level of induced systemic resistance compared with the potted experiment, as in Petri plates, higher levels of VOCs were available (Fig. 7B1 and B2). The decreased disease development in plants inoculated with VOCs-treated *Rsc* cells might be a consequence of the VOCs not only reducing pathogen growth but also restricting the movement of the pathogen into tobacco plant roots. VOCs from FZB42 and LSSC22 might be influenced in two positive ways: promoting plant growth and inhibiting pathogen growth, and thus inducing systemic resistance. Our results suggested that FZB42 and LSSC22 emit VOCs that can elicit plant defense mechanisms. The results showed the up-regulation of transcriptional expression of R gene *RRS1* along with the overexpression of *NPR1* and *EDS1* when exposed to VOCs after inoculation with *Rsc.* The over-expression of *RRS1* confirmed the induction of systemic resistance in tobacco as the *RRS1* is specifically the bacterial wilt resistance gene isolated from Arabdiopsis and detected in tobacco^34^. The R gene (RRS1-R) isolated from Nd-1 showed a significant enhancement of resistance when overexpressed in Col plants^57^,^58^. Overexpression of R gene, *RE-bw* in eggplant confirmed that the *RE-bw* was an important R gene against bacterial wilt. Furthermore, results showed the involvement of *RE-bw* in enrichment of SA content, ROS-scavenging enzymatic activities, cell wall, and lignin to minimize the *Rsc* colonization to roots. The up-regulation of transcriptional expression of *NPR1* and *EDS1* suggested that the induction of systemic resistance might be motivated by SA signaling path way as *EDS1* and *NPR1* are the main components of SA pathway^35^. The chemical compounds, BDH, 1,2-BITH and 1,3-BDN, are responsible for eliciting the defense response. Tobacco seedlings receive a sufficient amount of airborne chemical information to trigger ISR, as measured by the ability of the seedlings to resist infection. These results suggest that induced systemic resistance can be achieved without any contact between PGPRs and plants, indicating that VOCs might be involved normally in the process of induced systemic resistance^59^. These results are consistent with previous findings in which *Bacillus amyloliqefaciens* IN937a, *B. subtilis* GB03*, Escherichia coli* DH5a and *Pseudomonas fluorescens* 89B61 produced BDH, inducing systemic resistance in plants against pathogens and inhibited the growth of various pathogenic fungus mycelia and spore germination^21^,^60^,^61^. Similarly, benzothiazole inhibits mycelial growth^14^,^62^. BDH is an aromatic aldehyde with strong antimicrobial activity, but it is a generally regarded as safe (GRAS)-compound without any toxic effect in humans or on the environment^63^. In conclusion, our study showed the following novel results: (1) VOCs produced by FZB42 and LSSC22 inhibited colony growth, motility and negatively influenced the chemotaxis of *Rsc*, (2) caused morphological and ultra-structural abnormalities in *Rsc* cells, (3) decreased virulence levels both when *Rsc* cells were directly treated with VOCs and when plants inoculated with *Rsc* were exposed to VOCs, inducing systemic resistance, (4) altered the transcriptional expression of virulence related genes (T3SS, T4SS, EPS, motility and virulence regulator *PhcA*) and over-expressed genes related to wilt resistance and pathogen defense (5) BDH, 1,2-BIT, 1,3-BDN were not only the key inhibiting factors of *Rsc*, but also eliciting ISR. These results provide new insight into the inter-communication among the pathogenic bacteria *Rsc* and antagonistic bacteria, FZB42 and LSSC22 and tobacco plants in terms of the suppression of the wilt disease index and induction of systemic resistance in plants. This is the first report on the production and systemic resistance activity of VOCs derived from FZB42 and LSSC22 against bacterial wilt disease. VOCs produced by FZB42 and LSSC22 are good antibacterial compounds, providing an alternative to pesticides, and they can be used as an environmentally friendly biocontrol mechanism.

## Additional Information

**How to cite this article**: Tahir, H. A. S. *et al. Bacillus* volatiles adversely affect the physiology and ultra-structure of *Ralstonia solanacearum* and induce systemic resistance in tobacco against bacterial wilt. *Sci. Rep.* **7**, 40481; doi: 10.1038/srep40481 (2017).

**Publisher's note:** Springer Nature remains neutral with regard to jurisdictional claims in published maps and institutional affiliations.

## Supplementary Material

Supplementary Information
