# Supplementary material for: Bacillus volatiles adversely affect the physiology and ultra-structure of Ralstonia solanacearum and induce systemic resistance in tobacco against bacterial wilt
Source: Sci Rep. 2017 Jan 16;7:40481. doi: 10.1038/srep40481 (PMC5238454; doi:10.1038/srep40481)
Supplement: Supplementary Information [file srep40481-s1.pdf]

Supplementary Information

Bacillus volatiles adversely affect the physiology and ultra-structure of *Ralstonia solanacearum* and induce systemic resistance in tobacco against bacterial wilt

Hafiz Abdul Samad Tahir, Huijun Wu, Yuedi Niu, Qin Gu, Rong Hu, Xuewen Gao*

Department of Plant Pathology, College of Plant Protection, Nanjing Agricultural University, Key Laboratory of Integrated Management of Crop Diseases and Pests, Ministry of Education, Nanjing 210095, PR China

* Corresponding author: Xuewen Gao

Address: Nanjing Agricultural University, Weigang No.1, Nanjing 210095, PR China

Email: gaoxw@njau.edu.cn

Telephone/Fax: 86-25-8439526

Table S1: Bacteria used in this study

No	Strain	Antagonistic strain or pathogen	Accession NO
1	*Bacillis amyloliquefacians* FZB42	Antagonist	**NR075005.1**
2	*Bacillus cereus* NMSL88	Antagonist	**GU568190.1**
3	*Bacillus amyloliquefacians* NMSX4	Antagonist	**GU568185.1**
4	*Bacillus subtilis* FA26	Antagonist	**KY003098**
5	*Bacillus artrophaeus* LSSC22	Antagonist	**GU568193.1**
6	*Bacillus pumulis* GBSW19	Antagonist	**GU568202.1**
7	*Ralstonia solanacearum* TBBS1	Pathogen	**KY003096**

Table S2: Primers used in the study

No	Oligo Name	Sequence $5^{/}$ to $3^{/}$
1	*16sr RNA Rsc* TBBS1 (F)	AGTCCACGCCCTAAACGA
2	*16sr RNA Rsc* TBBS1 (R)	CGAAGGCACCAATCCATC
3	*Mot A* (F)	GTTTTCCATCATCCAAGC
4	*Mot A* (R)	GTGCCGAACAGACCCATC
5	*FliT* (F)	GACCAGCGCCCGGACACG
6	*FliT* (R)	CGCCGCAAGGCAACGACA
7	*Chew* (F)	CGGCGACCGTCATTGGCT
8	*Chew* (R)	GCGCTGCGGATCCTTGGA
9	*PhcA* (F)	GCAACGTCTGCCTTTTCACT
10	*PhcA* (R)	CGCTGTCATGTGCATCTTCT
11	*HrpB* (F)	AGACCAAGGTGGAAGTCGTG
12	*HrpB* (R)	CGTCTTGCATGTAGCTGGTG
13	*EpsA* (F)	TTCCTCTGACCCAAGGAATG
14	*EpsA* (R)	ATCAAAGGTGTAGCCGTTGG
15	*EpsB* (F)	GGCGTTGTCCTAGGTGTCAT
16	*EpsB* (R)	CGCTTCGATAGATGGGTCAT
17	*EpsC* (F)	ACGGTGACACTTCAACCACA
18	*EpsC* (R)	GCCATGGGCTGTACAAGTTT

19	*EpsD* (F)	CAGCCGAGATGTGCAAACTA
20	*EpsD* (R)	GGGGCAGCATCTACGATAAA
21	*EpsE* (F)	TACCGGCCACACTAGGAAAC
22	*EpSE* (R)	TTTCGCCTGCTAGCAAAAAT
23	*EpsF* (F)	GTTTCTCATCACGGCGTTTT
24	*EpsF* (R)	GGCGAAGGCTATGCTAGATG
25	*EpsP* (F)	CGTGAGATACAGGCGAGACA
26	*EpsP* (R)	GTCTTCGAACGCCATCATTT
27	*Awr1*(F)	ACGTTTCCACGCATAACTCC
28	*Awr1* (R)	GTCTGGGTGGCAAAAAGTGT
29	*Awr3* (F)	CTCACGCATTCCTACAAGCA
30	*Awr3* (R)	CCAGTCTGCTCAGGTGACAA
31	*Awr5* (F)	CACATGGCGGAGAGATTTTT
32	*Awr5* (R)	TCGTAGACGTACGCCTGTTG
33	*PilQ* (F)	CAGGGACAAAACTTGGTCGT
34	*PilQ* (R)	CGGAGTCGGTAGCTCTCATC
35	*RRS1*(F)	ATGAGAAAGAGGCTCGTCAA
36	*RRS1*(R)	ACCACAACCCTCAAGCAGTT
37	*NPR1*(F)	CTGGAGCAAGCAGAAAG
38	*NPR1*(R)	TCATACGCAAATCATCG
39	*EDS1*(F)	GAG TAT CAG ACC AAG TGT GAT ATC CG
40	*EDS1*(R)	GCT GAG GTG GGA GTG TTT TCC ACC

Table S3: VOC profile of *Bacillus amyloliquefacians* FZB42 and *Bacillus artrophaeus* LSSC22

Bacillus strain	RT (min)	Relative peak area (%)	Compound	Abbreviation used	Degree of inhibition
***Bacillus amyloliquefacians* FZB42**	4.567	3.829	Silanediol, dimethyl	SDD	_
	5.618	3.574	1,2-Benz isothiazol-3(2H)-one	1,2-BIT	+++
	6.963	1.865	Benzeneacetamide	BAM	++
	7.631	2.573	Oxime,methoxy-phenyl	OMP	NT
	7.787	3.494	(1R)-2,6,6-Trimetyhlbicyclo[3.1.1]hept-2-ene	TMB	+
	8.207	1.557	Benzoic acid, 2-formyl-4,6-dimethoxy-,8,8-dimethoxyoct-2-yl	BA	+
	8.949	1.695	Benzaldehyde	BDH	+++
	9.543	2.277	Sulfurous acid, cyclohexylmethyl isobutyl ester	SCE	_
	10.148	1.406	6-Tridecen, 2,2,4,10,12,12-hexamethyl-7-(3,5,5-trimrthylhexyl)-	6 -THT	NT
	10.304	2.116	2-Undecanethiol, 2-methyl	2-UT,2-M	_
	10.734	6.490	Dodecane , 1-fluoro	DCF	++
	12.262	1.695	Dodecane	DCN	++
	14.421	2.380	Phenol, 2-(1,1-dimethyl)-5-methyl-	PH	_
***Bacillus artrophaeus* LSSC22**	1.301	1.073	1,3 –Butadiene	1,3-BDN	++
	5.631	5.995	1,2-Benz isothiazol-3(2H)-one	1,2-BIT	+++
	7.729	2.245	(1R)-2,6,6-Trimetyhlbicyclo[3.1.1]hept-2-ene	TMB	+
	8.764	3.483	Benzoic acid	BA	+
	10.487	2.204	1-octyn-3ol, 4- ethyl-	1,OTN	++
	10.712	8.354	Dodecane , 1-fluoro	DCF	++
	11.575	1.491	Undecanal, 2-methyl	UDM	++
	12.264	2.768	Dodecane	DCN	++
	14.413	2.694	Phenol, 2-(1,1-dimethylethyl)-6-methyl	PH	_
	16.669	1.213	Cyclohexene, 3-(1,5-dimethyl-4-hexenyl)-6-methylene-	CHN	NT

Minor air-peaks were excluded from the total analysis representing ≤1% of the total

area. Similar compounds found in both inoculated and non-inoculated MS medium were also not included. **RT**, retention time, — no inhibition, + ≤10% inhibition rate, + + 10-30 % inhibition rate, + + + More than 30 % inhibition, **NT** Not tested

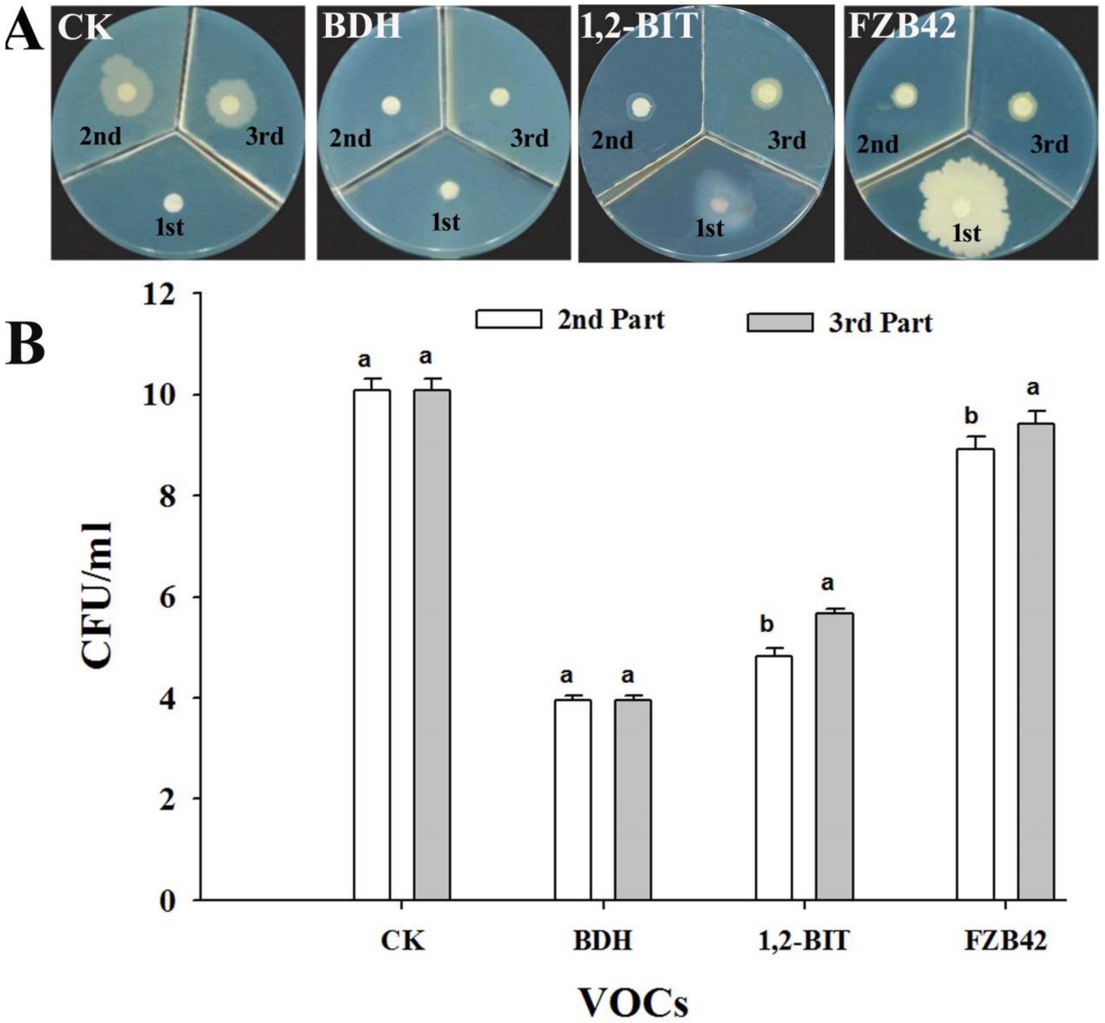

Fig S1 Effect of the concentration of VOCs The experiment was conducted in three partition plates with completely sealed portions without any air movement except holes in two walls of the partitions. An arrangement was made so that VOCs could move from the first partition, encompassing *Bacillus* or a synthetic chemical, to the second partition and then from the second partition to the third partition. The second and third partition both were inoculated with 10 µl of *Rsc* (18-24h) culture. The whole experiment was repeated three times, including three replicates per experiment. Letters

above error bars represent significant differences according to Duncan’s multiple-range test (P=0.05) using SPSS software (SPSS, Chicago, IL).

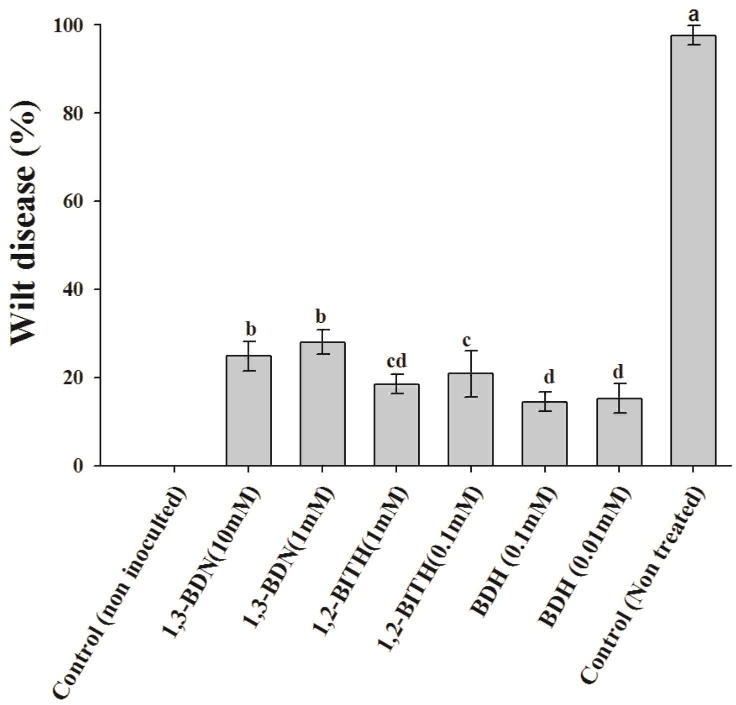

Fig S-2 BDH, 1,2-BITH and 1,3-BDN reduced the virulence of *Ralstonia solanacearum* and induced systemic resistance I-plates prepared with one-half-strength Murashige and Skoog solid medium, and 5-6-day-old emerging tobacco seedlings (seven seedlings/plate) were dipped in the suspension of *Rsc* (10^7 CFU/mL) cells and transplanted into one compartment. In the non-inoculated control, the roots were dipped in sterile water. Chemicals (10mM and 1mM 1,3-BDN, 1mM and 0.1mM 1,2-BITH while 0.1mM and 0.01mM BDH) were used in the other compartment. Wilt symptoms were observed after 7 days of inoculation, and the data were recorded. Error bars indicate standard deviations of the means. Different letters above error bars represent significant differences according to Duncan’s multiple-range test (P=0.05) using SPSS software (SPSS, Chicago, IL).
